# Effect of Quality Control Circle Activity Nursing Combined with Respiratory Function Exercise Nursing on Patients with Esophageal Cancer

**DOI:** 10.1155/2022/8607760

**Published:** 2022-09-17

**Authors:** Hairu Yu, Sha Li, Shasha Shi

**Affiliations:** ^1^Department of Thoracic Cancer, Hubei Cancer Hospital, Tongji Medical College, Huazhong University of Science and Technology, Wuhan, China; ^2^Department of Gastroenterology, Wuhan Third Hospital, No. 241 Shouyi Road, Wuchang, Wuhan, China

## Abstract

**Objective:**

To explore the effect of quality control circle activity nursing combined with respiratory function exercise nursing on esophageal cancer patients' immune function and nutritional status.

**Methods:**

The clinical case data of 119 esophageal cancer patients admitted to our hospital were selected as the research objects from May 2019 to July 2021. They were divided into the quality control circle activity care group (QCCAC) (9 cases dropped due to incomplete case data, n= 50) and respiratory function exercise care group (10 cases dropped due to incomplete case data, n=50) by the random number table method, the respiratory function exercise care group was treated with respiratory function exercise care, while the QCCAC group was treated with QCCAC. Changes in lung function, immune function, and nutritional status before and after nursing were compared in the two groups of patients.

**Results:**

Before nursing, there was no significant difference in pulmonary function indexes, immune function indexes, and the level of nutritional status indicators between the two groups (*P* > 0.05). After nursing, the finger pulse oxygen saturation, vital capacity (VC), respiratory rate, forced vital capacity (FVC), deep inspiratory volume (IC), and maximum ventilation (MVV) of the QCCAC improved, and the QCCAC group was significantly higher than the respiratory function exercise care group (*P* < 0.05). After nursing, the QCCAC's CD3+, CD4+, IgG, IgM, and IgA levels increased, and the QCCAC group was significantly higher than the respiratory function exercise care group. The CD8+ level decreased, and the QCCAC was lower than the respiratory function exercise care group (*P* < 0.05). After continuous nursing, the QCCAC's D-lactic acid, DAO, FFMI, Hb, ALB, PA, and other nutritional evaluation indexes all increased, and the QCCAC was significantly higher than the respiratory function exercise care group (*P* < 0.05).

**Conclusion:**

Quality control circle activity nursing combined with respiratory function exercise nursing can effectively improve the immune function, respiratory function, and nutritional status of esophageal cancer patients.

## 1. Introduction

Esophageal cancer is one of the most common malignant tumors in China and is the malignant tumor with the fourth highest fatality rate [1]. Esophageal cancer belongs to the body's esophageal epithelial malignant lesions, accounting for 2% of the incidence of all malignant tumors, and the number of patients who die of esophageal cancer worldwide each year reaches about 200,000, and China has a high incidence of esophageal cancer [2]. At present, the main treatment for esophageal cancer is surgery, supplemented by chemotherapy. Cancer itself has a huge psychological impact on patients, coupled with the symptoms caused by tumors, adverse reactions caused by surgery, radiotherapy, and chemotherapy, which bring a huge burden to patients [3]. Patients are often accompanied by negative emotions such as anxiety and depression, which affect the occurrence and development of tumors and adversely affect the quality of life [4]. With the transformation of the medical model to the biological-psychological-social model, paying attention to the psychological function of cancer patients and adjusting the patients' negative emotions have become an important aspect of cancer treatment and care [5]. In recent years, studies have pointed out that carrying out quality control circle activities in the nursing field can further improve the effectiveness of nursing, improve the quality of nursing services, and encourage patients to cooperate with clinical medical work [6]. Quality control circle activities are used in the rapid rehabilitation of surgical care patients after surgery. The corresponding rehabilitation exercise recovery plan is reasonably formulated based on the patient's condition. The patient is guided and supervised quantitatively and targeted to improve patient compliance and exercise science, thereby improving the prognosis [7]. Cognitive behavioural therapy is a short-term psychotherapy that can eliminate bad emotions and behaviours by changing negative cognition and thoughts or beliefs and behaviours [8]. This therapy is widely used to treat anxiety, depression, insomnia, and other mental and psychological diseases [9]. In recent years, cognitive behavioural therapy has been applied to patients with malignant tumors and has achieved certain results. This study included 119 esophageal cancer patients admitted to our hospital in the past two years as the observation object, aiming to explore the effect of quality control circle activity nursing combined with respiratory function exercise nursing on the respiratory function exercise of esophageal cancer patients to provide esophageal cancer patients new nursing ideas. The report is as follows.

## 2. Materials and Methods

### 2.1. General Information

From May 2019 to July 2021, the clinical case data of 119 esophageal cancer patients admitted to our hospital were selected as the research objects. They were divided into the quality control circle activity care group (QCCAC) and respiratory function exercise care group by the random number table method. Nine patients were excluded from the study due to incomplete clinical data, and 50 were finally included. In the respiratory function exercise care group, 10 cases were dropped due to incomplete case data, and 50 cases were finally included. The two groups of patients had no significant difference in general information such as age, gender, body mass index (BMI), type of esophageal cancer, disease course, whether there is a nutritional risk, prognosis, survival time, etc. (*P* > 0.05), and they are comparable, as shown in Table 1. During the 6-month follow-up period, 3 cases died in the QCCAC, 6 cases were lost to follow-up, and 50 cases were finally completed; 3 cases died in the respiratory function exercise care group, 7 cases were lost to follow-up, and 50 cases were finally completed. Inclusion criteria were as follows: (1) all patients underwent chest computed tomography (CT), esophageal barium meal or electronic gastroscopy before treatment, and all had clear pathological diagnoses [10]; (2) newly treated primary esophageal cancer and received radical radiotherapy for the first time; (3) KPS score>70 with the independent reading ability or completed the required questionnaire with the help of the investigator and the patient's age is <75 years old; (4) patients and their families agreed to participate in this study and signed informed consent forms; and (5) patients are eligible for post-follow-up investigation. Exclusion criteria were as follows: (1) patients with a history of other malignant tumors and patients whose surgical plan was changed; (2) patients with mental illness who participated in other similar research during the hospitalization; (3) patients with severe gastrointestinal dysfunction and incomplete clinical data; (4) those who cannot tolerate surgical treatment; (5) patients with cardiac, hepatic, and renal insufficiency; and (6) people with other diseases.

### 2.2. Method

#### 2.2.1. Respiratory Function Exercise Care Group

The respiratory function exercise care group used respiratory function exercise care and abdominal breathing training: relaxing all auxiliary respiratory muscles, sinking the abdomen when exhaling, and placing a sandbag on the upper abdomen to further increase abdominal pressure. When exhaling, the upper abdomen resists the pressure of the sandbag and slowly bulges the abdomen. When exhaling, the abdomen slowly sinks, and it is repeated many times. Starting from each exercise for 5 minutes, the time is gradually extended until you can breathe steadily for 2 hours with the sand bag placed. The breathing rate changes from fast to slow, eventually reaching about 9 breaths per minute. The sandbag increases from light to heavy, from 2 kg to 5 kg. Generally, patients exercise for 1 or 2 weeks before surgery, while those who are elderly have a poor respiratory function and have a larger scope of surgery exercise for 3 or 4 weeks before surgery and can be extended appropriately. Effective cough training: instruct the patient to take a deep breath and hold their breath, close the glottis, raise the diaphragm to increase the intrapleural pressure, contract the intercostal muscles, then cough, and open the glottis to flush out the gas or sputum. The patient repeats the exercises until they master it.

#### 2.2.2. QCCAC

The implements of QCCAC, (1) establishes a quality control circle activity group. The team members include the head nurse, the head nurse, and the head nurse in charge of the team. The ward nurses are divided into 4 groups, the head nurse manages each group, and the head nurse assigns the detailed work flow. After the head nurse assigns the work, the head nurse assigns the work to the group members and provides professional guidance. The task is arranged from top to bottom, and effective feedback is obtained during the work (recording the problems in work) and then reported from the bottom to the top. Discuss and solve in a unified way. (2) Develop a reasonable solution plan. After the activity time is determined, nurses are arranged in batches to learn the knowledge of the quality control circle. Based on the “Chinese Hospital Quality Control Circle Operation Manual,” according to the theme of this activity, patients with esophageal cancer after surgery are guided to perform respiratory function exercises properly and formulate regular and quantitative breathing. The specific content of the exercise is as follows: 6 to 10 hours after anesthesia: maintain a semirecumbent position on the bed and move your legs twice for 5 minutes each time, after completion of aerosol inhalation, cough and expectorant, take 2 deep breaths for 5 minutes each time. Day 1 after surgery: Move your legs and legs 4 times on the bed for 5 minutes, continue to sit on the bed, cough and cough up sputum after nebulized inhalation, breathe deeply 6 times for 5 minutes each time, stand on the edge of the bed and move 2 times, 100 meters each time. The day after the operation to the discharge: move your legs 6 times on the bed for 5 minutes, sit on the bed all the time, after the aerosol inhalation, cough and sputum production are completed, take 6 deep breaths for 10 minutes each time, stand at the edge of the bed and move 6 times, each time moving 200 ~ -500m. The activity distance gradually increases with time and status. At the same time, improve the nursing staff's professional knowledge of breathing exercise training and guidance ability. After the patient is admitted to the hospital, patiently, calmly, and orderly give the patient breathing exercise guidance to make it clear the importance of respiratory function for rapid postoperative recovery and help them master correct breathing techniques to improve the correct rate of breathing exercises and reduce or avoid pulmonary complications. (3) Nutritional care: patients mainly eat porridge, vegetable soup, and other foods during radiotherapy. They eat sugar-free porridge for breakfast and dinner and vegetable soup (greens, carrots, seaweed, etc.) in the morning, noon, and afternoon. Instruct patients to eat at least 5 times a day, add 1000 ml of water before meals, add 1000 ml between two meals, and drink ≥5000 ml of water daily to prevent vomiting during chemotherapy.

### 2.3. Observation Indicators

(1) Pulmonary function monitoring: using the Master ScreenDiffusion machine from JAEGER, Germany, professional physicians will perform pulmonary function tests on the first day of hospitalization and 1 day before surgery and observe and record before exercise (the day of admission) and after exercise (1 day before surgery). Changes in lung function indicators: observation items include vital capacity (VC), forced vital capacity (FVC), deep inspiration (IC), and maximum ventilation (MVV). Finger pulse oxygen saturation was measured by clamping the end of the patient's finger with the finger pulse oxygen saturation monitor of NONINMEDI-CAL. (2) Immune function: before and after 6 months of nursing, peripheral venous blood samples of patients were collected, and use FACS calibur flow cytometer and supporting reagents were to detect and analyze CD3+, CD4+, CD8+ and use flow cytometer to detect and analyze [11]. All operations are carried out in strict accordance with the instrument manual. The immunoglobulin IgG, IgM, and IgA content were detected by enzyme-linked immunosorbent assay, and all operations were performed per the kit instructions [12]. (3) Nutritional status: measure D-lactic acid levels, diamine oxidase (DAO) levels, fat-free body mass index (FFMI), hemoglobin (Hb), albumin (ALB), prealbumin (PA), and other individual nutritional status evaluation indicators [13].

### 2.4. Statistical Methods

SPSS25.00 statistical software was used to analyze the data. Measurement data were expressed as mean ± standard deviation (±*s*), and a *t*-test was performed between groups; a rank sum test was performed for non-normal distribution, and count data were expressed as a percentage (%). *χ*^2^ test was performed, and *P* < 0.05 indicated that the difference was statistically significant.

## 3. Results

### 3.1. Comparison of Lung Function Indicators between the Two Groups

Before nursing, there was no significant difference in pulmonary function indexes between the two groups of patients (*P* > 0.05). After nursing, the QCCAC's finger pulse oxygen saturation, vital capacity (VC), respiratory rate, forced vital capacity (FVC), deep inspiratory volume (IC), and maximum ventilation (MVV) were improved, and the QCCAC was better than the respiratory function exercise care group. The difference was statistically significant (*P* < 0.05) (see Table 2).

### 3.2. Comparison of the Levels of Immune Function Indicators

Before nursing, there was no statistical difference in the immune function indexes between the two groups (*P* > 0.05). After nursing, the QCCAC's CD3+, CD4+ and IgG, IgM, and IgA levels increased. The QCCAC group was higher than the respiratory function exercise care group. The CD8+ level decreased, and the QCCAC group was higher than the respiratory function exercise care group; the above differences were statistically significant (*P* < 0.05) (see Tables 3 and 4).

### 3.3. Comparison of Nutritional Status Indicators

Before nursing, there was no statistically significant difference in the level of nutritional status indicators between the two groups (*P* > 0.05). After nursing, the QCCAC's D-lactic acid, DAO, FFMI, Hb, ALB, PA, and other nutritional evaluation indexes all increased. The QCCAC groupwas higher than the respiratory function exercise care group; the difference was statistically significant (*P* < 0.05) (see Tables 5 and 6).

## 4. Discussion

With the transformation of medical models, clinicians have realized that the purpose of modern medicine is not only to treat patients' diseases and improve patients' organ functions but also to pay attention to patients' mental state and improve patients' quality of life [14]. Esophageal cancer is a common clinical malignant tumor. There is currently no specific treatment plan. Only treatment can be used to control the patient's condition, remove the patient's tumor lesions, improve the patient's postoperative survival rate, and improve the quality of life [15]. However, after research and analysis of literature, it is found that esophageal cancer patients' postoperative quality of life is significantly lower than that of ordinary people [16]. Among esophageal cancer patients, the elderly are the majority. The elderly are not well-educated, have weak knowledge structures, and have varying doubts and resistance to clinical treatment. Some patients do not cooperate with clinical treatment due to economic conditions, which makes the overall treatment effect unsatisfactory. The prognosis is poor [17]. Most patients with esophageal cancer have difficulty getting out of bed and coughing after esophageal cancer surgery. Most patients with esophageal cancer still need to return to the hospital for treatment after discharge. The model of nursing care ensures that medical resources are effectively and reasonably used to strengthen nursing management, improve patient compliance with treatment, and encourage them to exercise respiratory function, thereby improving the prognosis [18].

After nursing, the finger pulse oxygen saturation, vital capacity (VC), respiratory rate, forced vital capacity (FVC), deep inspiratory volume (IC), and maximum ventilation (MVV) of the QCCAC improved and the QCCAC was better than the respiratory function exercise care group, indicating that quality control circle activity nursing combined with respiratory function exercise nursing can improve the respiratory function of patients with esophageal cancer. The quality control circle activity was first applied in corporate management. The main purpose of the activity is to encourage all employees to participate in the activity, improve their collective sense of honor, and encourage employees to maintain and improve the products produced by the company, to improve their work efficiency and quality. However, because the quality control circle activity is a very widely adaptable activity, more and more managers realize the high efficiency of its application, so the quality control circle activity covers more and more fields [19]. In recent years, studies have pointed out that carrying out quality control circle activities in the nursing field can further improve the effectiveness of nursing, improve the quality of nursing services, and encourage patients to cooperate with clinical medical work [20]. Quality control circle activities are used in the rapid rehabilitation of surgical care after surgery. The corresponding rehabilitation exercise recovery plan is reasonably formulated based on the patient's condition. The patients are guided and supervised quantitatively and targeted to improve patient compliance and exercise science, thereby improving prognosis [21]. Studies have pointed out that the application of quality control circle activities in the rapid recovery surgical care of patients after esophageal cancer can effectively improve the compliance and the accuracy of breathing exercises, effectively enhance the status of patient's lung function, and significantly shorten the discharge time [22].

After nursing, the CD3+, CD4+ and IgG, IgM, and IgA levels of QCCAC were increased, and the QCCAC group was higher than the respiratory function exercise care group. The CD8+ level decreased, and the QCCAC group was lower than the respiratory function exercise care group. Quality control circle activity nursing combined with respiratory function exercise nursing can improve the immune function of esophageal cancer patients effectively. The immune function of tumor patients is often suppressed. Malnutrition can lead to low immune function. Surgical stress will aggravate the immunosuppressive state, which is manifested as a decrease in the circulating helper lymphocyte subsets CD4+ and a relative increase in the inhibitory lymphocyte subsets CD8+ and CD4+/CD8. The proportion decreases, and the activity of NK cells decreases [23].

After nursing in this study, the QCCAC's D-lactic acid, DAO, FFMI, Hb, ALB, PA, and other nutritional evaluation indexes all increased, and the QCCAC group was higher than the respiratory function exercise care group. Quality control circle activity nursing combined with respiratory function exercise nursing can improve patients' nutritional status. Bacteria produce d-lactic acid in the gastrointestinal tract, and mammals have no fast-metabolizing enzyme system. The detection of blood D-lactic acid levels can reflect changes in intestinal mucosal permeability in time [24]. DAO in the small intestinal mucosa's upper villi can reflect the small intestine's structure and function. DAO activity in peripheral blood is stable, so its changes in peripheral blood can be measured non-invasively to reflect the state of intestinal mucosa [25].

In summary, the quality control circle activity nursing combined with respiratory function exercise nursing can effectively improve the immune function, respiratory function, and nutritional status of patients with esophageal cancer. Clinical application is recommended. In addition, since this study only observed the results of nursing care without long-term evaluation and the sample size is small, clinical applications should also be considered.

## Figures and Tables

**Table 1:** Comparison of general information between QCCAC and respiratory function exercise care group (±*s*).

Group	Age (years)	Gender	BMI	Course of disease (years)
		Male/female		
QCCAC (*n* = 50)	58.35 ± 9.43	28/22	23.26 ± 3.88	6.54 ± 1.43
Respiratory function exercise care group (*n* = 50)	59.41 ± 10.65	26/24	22.79 ± 4.12	6.91 ± 1.15
*t/χ * ^2^	0.527	0.161	0.587	−1.426
*P*	0.599	0.688	0.558	0.157

**Table 2:** Comparison of lung function indexes (±*s*).

Group	Finger pulse oxygen saturation (%)		VC (L)		Respiratory rate (%)	
	Before care	After care	Before care	After care	Before care	After care
QCCAC (*n* = 50)	96.23 ± 10.23	98.77 ± 2.64	3.16 ± 0.81	3.76 ± 0.97	19.82 ± 5.04	14.47 ± 0.43
Respiratory function exercise care group (*n* = 50)	96.46 ± 10.46	96.81 ± 2.19^*∗∗∗*^	3.14 ± 0.31	3.26 ± 0.25^*∗∗∗*^	19.44 ± 4.91	16.09 ± 0.48^*∗∗∗*^
*t*	0.111	4.040	0.163	3.530	0.382	−17.775
*P*	0.912	<0.001	0.871	<0.001	0.703	<0.001

*Group*	*FVC (L)*		*IC (L)*		*MVV (L)*	
	*Before care*	*After care*	*Before care*	*After care*	*Before care*	*After care*

QCCAC(*n* = 50)	2.33 ± 0.23	2.97 ± 0.64	2.26 ± 0.51	2.76 ± 0.77	81.51 ± 3.42	87.23 ± 8.57
Respiratory function exercise care group(*n* = 50)	2.36 ± 0.46	2.41 ± 0.19^*∗∗∗*^	2.14 ± 0.41	2.46 ± 0.25^*∗∗*^	81.57 ± 3.81	83.06 ± 7.63^*∗*^
*t*	0.412	5.931	1.297	2.620	-0.082	2.570
*P*	0.681	<0.001	0.198	0.010	0.935	0.012

*Note.* ^*∗*^ *P* < 0.05, ^*∗∗*^*P* < 0.01, and ^*∗∗∗*^*P* < 0.001 vs. before care.

**Table 3:** Comparison of CD3+, CD4+, and CD8+ levels (±*s*).

Group	CD4^+^ (%)		CD8^+^ (%)		CD3^+^ (%)	
	Before care	Aftercare	Before care	Aftercare	Before care	Aftercare
QCCAC (*n* = 50)	34.63 ± 7.83	38.87 ± 8.61^*∗*^	29.34 ± 2.95	27.01 ± 3.42^*∗*^	61.23 ± 8.57	66.11 ± 8.59^*∗*^
Respiratory function exercise care group (*n* = 50)	32.76 ± 7.51	35.41 ± 7.31^*∗*^	30.26 ± 3.64	28.57 ± 3.81^*∗*^	59.06 ± 7.63	62.53 ± 8.36
*t*	1.188	2.109	1.355	2.099	1.303	2.048
*P*	0.238	0.038	0.179	0.039	0.196	0.043

*Note*. ^*∗*^*P* < 0.05 vs. before care.

**Table 4:** Comparison of IgG, IgM, and IgA levels (±*s*).

Group	IgG (g/L)		IgM (g/L)		IgA (g/L)	
	Before care	Aftercare	Before care	Aftercare	Before care	Aftercare
QCCAC (*n* = 50)	12.95 ± 4.35	15.82 ± 5.04^*∗*^	1.17 ± 0.43	1.44 ± 0.46^*∗*^	2.08 ± 0.12	2.61 ± 0.19
Respiratory function exercise care group (*n* = 50)	13.27 ± 4.46	13.44 ± 4.91	1.09 ± 0.48	1.26 ± 0.41^*∗*^	2.11 ± 0.14	2.49 ± 0.15^*∗∗∗*^
*t*	0.354	2.331	1.178	2.012	1.122	3.412
*P*	0.724	0.022	0.242	0.047	0.265	<0.001

*Note.* ^*∗*^ *P* < 0.05, ^*∗∗*^*P* < 0.01, and ^*∗∗∗*^*P* < 0.001 vs. before care.

**Table 5:** Comparison of D-lactic acid, DAO, and FFMI levels before and after surgery (±*s*).

Group	D-Lactic acid (mg/L)		DAO (U/mL)		FFMI (kg/m^2^)	
	Before care	Aftercare	Before care	After care	Before care	Aftercare
QCCAC (*n* = 50)	11.89 ± 6.38	13.62 ± 7.13^*∗*^	2.76 ± 4.45	4.97 ± 4.8^*∗*^	15.22 ± 3.21	18.82 ± 3.74^*∗∗*^
Respiratory function exercise care group (*n* = 50)	10.21 ± 5.41	16.57 ± 7.22^*∗*^	2.32 ± 3.96	5.69 ± 4.78^*∗*^	14.72 ± 2.83	16.71 ± 3.66^*∗∗*^
*t*	1.383	-2.003	0.647	-2.310	0.712	2.779
*P*	0.170	0.048	0.519	0.023	0.478	0.007

^*∗*^ *P* < 0.05, ^*∗∗*^*P* < 0.01, and ^*∗∗∗*^*P* < 0.001 vs. before care.

**Table 6:** Comparison of Hb, ALB, and PA levels before and after surgery (±*s*).

Group	Hb (g/L)		ALB (g/L)		PA (mg/L)	
	Before care	After care	Before care	After care	Before care	After care
QCCAC (*n* = 50)	116.23 ± 31.23	148.77 ± 33.64^*∗*^	35.26 ± 7.81	43.76 ± 7.97^*∗∗*^	278.15 ± 79.41	347.46 ± 83.64^*∗*^
Respiratory function exercise care group (*n* = 50)	119.46 ± 29.46	131.81 ± 30.19^*∗*^	34.94 ± 8.01	39.26 ± 8.25^*∗∗*^	277.36 ± 77.63	309.83 ± 79.61^*∗*^
*t*	0.518	2.117	0.197	2.704	0.049	2.245
*P*	0.606	0.012	0.844	0.008	0.961	0.027

*Note*. ^*∗*^*P* < 0.05, ^*∗∗*^*P* < 0.01, and ^*∗∗∗*^*P* < 0.001 vs. before care.

## Data Availability

The datasets used and analyzed during the current study are available from the corresponding author upon reasonable request.
